# Transluminal Nd: YAG laser embolysis for branch retinal artery occlusion: Case report

**DOI:** 10.1097/MD.0000000000027984

**Published:** 2022-03-11

**Authors:** Wei Zhang, Ning An, Xi Zhang, Wei Gu, Xiaoyan Peng

**Affiliations:** a Department of the Ophthalmology, Beijing Aier Intech Eye Hospital, Beijing, China; b Department of the Ophthalmology, Beijing Tongren Eye Hospital, Capital Medical University, Beijing, China.

**Keywords:** branch retinal artery occlusion, transluminal Nd:YAG laser embolysis, treatment outcome

## Abstract

**Rationale:**

Central retinal artery occlusion and branch retinal artery occlusion (BRAO) result in partial or complete retinal ischemia and sudden loss of vision; to date there is no effective therapy for central retinal artery occlusion and BRAO. Transluminal Nd:YAG laser embolysis (TYE) could represent a therapeutic approach for retinal vascular occlusive diseases.

**Patient concerns:**

We report 2 cases with BRAO, 1 with inferor-temporal and 1 with superor hemiretinal BRAO. All the patients complained of a history of sudden blurry vision and impaired visual field and had a visible embolus within the intravascular, all of them treated with TYE, the laser applications being delivered directly to the embolus.

**Diagnosis:**

The diagnosis was based on the results from color retinography, optical coherence tomography and visual field testing. Fundus fluorescein angiography clearly indicated the location of retinal artery occlusion.

**Interventions:**

The patients’ symptoms could not be relieved after dilating the blood vessels in the eye, lowering intraocular pressure, massaging the eyeball, and inhaling oxygen. Informed consent was obtained from the patient for TYE and the patients were referred for this procedure.

**Outcomes:**

Upon the successful competition of the TYE procedure the embolus was removed completely, restoring the blood flow in the intraocular vessels and improving significantly the patients’ visual acuity.

**Lessons:**

World-wide experience with TYE is still limited, but the technique seems feasible for the treatment of RAO caused by visible emboli on the optic disc surface and the posterior pole of the fundus oculi.

## 1 Introduction

Branch retinal artery occlusion (BRAO) can be caused by platelet fibrino emboli, cholesterol emboli and calcific emboli, typically located on arteriolar bifurcations or areas of vascular stenosis.^[1]^ A rather frequent occurrence of retinal vascular pathologies is temporal artery branch occlusion (superior or inferior) caused by emboli located at the arterial branch's emergence from the optic nerve papilla. Blood flow blockage through the artery causes partial or complete retinal ischemia, with sudden loss of visual acuity and visual field (VF) impairment.^[2]^ The ocular fundus exam reveals sectorial clouding of the retina and most of the time, allows for the viewing of the translumenal embolus. The fluoresceine angiography exam shows either a delay or complete absence of dye material filling the affected blood vessel.^[3]^ Histopathologically, retinal artery branch occlusion is characterized by intracellular edema in the internal retinal layers with loss of cells (over several months) extending from the nerve fiber layer to the inner nuclear layer.^[4]^ However, medical literature still lacks a well-established treatment for such conditions.

Opremcak and Benner^[1]^ introduced the idea of using photo-disrupting Nd:YAG laser the selective lysing of intravascular solid embolus without damaging vascular walls. The aforementioned authors presented in April of 2002, 2 surprisingly efficient solved cases, both anatomically and functionally, through transluminal Nd:YAG laser embolysis (TYE). In both cases immediate clearing of the embolus was noted alongside full recovery of retinal blood flow and a relatively fast recovery of visual function (1–2 weeks). Other authors have reported favorable results using TYE. However, there are also some reports on the possible serious complications caused by TYE.^[2–5]^

Currently, there are still no unified clinical operation guidelines for TYE. World-wide experience with TYE is still reduced, but the technique seems feasible in treating BRAO and also central retinal artery occlusion (CRAO) caused by visible emboli.^[6]^ We have successfully cured 2 BRVO patients recently, and accumulated some treatment experience. Ethics Committee of Beijing Aier intech Eye Hospital approved the TYE procedure for the treatment of retinal artery occlusion. TYE is considered to be a safely and effectively therapeutic approach which can be used in certain situations. Herein, we report the cases of 2 patients who provided informed consent for publication.

## 2 Case presentation

### 2.1 Patient 1

**Figure 1:**
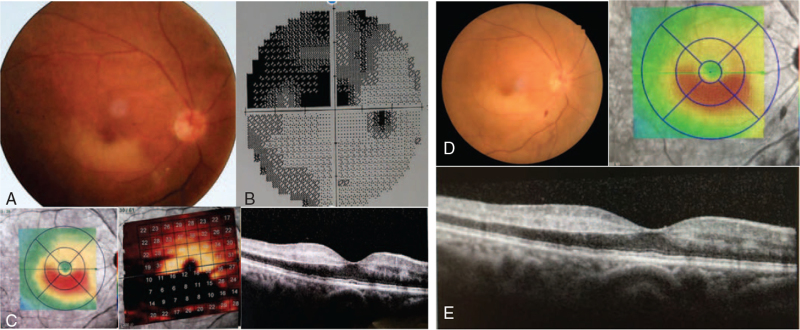
A: Color retinography showed inferior temporal artery branch occlusion, retinal oedema in the lower macular region, visible embolus in the first branch of the blood vessel from the papilla. B: VF showed a superior arcuate scotoma. C: OCT showed increased reflectivity and thickness of the inner retina in the surrounding inferior area, the inferior of retinal ganglion cells atrophy. D: Color retinal photography showed reduced retinal edema, a small amount of bleeding was visible at the laser point on the second day after laser treatment. E: OCT showed reduced high reflectivity in the inner layer of the retina on the second day after laser treatment. OCT = optical coherence tomography. VF = visual field.

A 75 years old male patient complained of a history of sudden blurry vision and superior altitudinal VF defect in his right eye which occurred 5 days ago. At the time of ocular examination, his best corrected visual acuity (BCVA) was 16/200 in the right eye and 20/20 in the left eye. Intraocular pressure (IOP) and slit-lamp examination was normal in both eyes. The patient had no history of ocular disease, surgery, or trauma to either eye. The patient had well-controlled type 2 diabetes, hyperlipidemia for 20 years, a history of cerebrovascular infarction for 9 years, and heart stent surgery 2 years prior. After thorough investigations including color retinography, optical coherence tomography (OCT) and VF testing, the diagnosis was inferior temporal artery branch occlusion with retinal oedema in the lower macular region, with a visible embolus in the first branch of the blood vessel from the papilla (Fig. 1A). VF showed a superior arcuate scotoma (Fig. 1B), and the inferior of retinal ganglion cells atrophy, and increased reflectivity and thickness of the inner retina were observed in the surrounding inferior area on OCT (Fig. 1C). The fundus of the left eye was normal.

After dilating the blood vessels, lowering IOP, massaging the eyeball, and oxygen administration, the patient's right eye symptoms could not be relieved. After obtaining an informed consent for TYE for BRAO, the patient was referred for TYE procedure, which was successfully completed, thus breaking the embolus and completely restoring blood flow.

The right eye was anesthetized with topical drops. A fundus contact lens (Idrees Mid Vitreous, VOLK) was used to focus the Nd:YAG laser (**Ellex UltraQ Reflex**, **AU**) on the embolus within the retinal artery. A 0.5 mJ pulse was initially delivered directly to the embolus for embolysis. During the procedure a small amount of blood flowed from the blocked artery, and the bleeding stopped after proper pressure with the laser lens. During the embolysis and opening of the arterial wall, some parts of the embolus could have been passed into the vitreous. On the second day after laser treatment, the patient's right eye BCVA improved to 16/20, color retinal photography showed reduced retinal edema, a small amount of bleeding was visible at the laser point (Fig. 1D), and OCT showed reduced high reflectivity in the inner layer of the retina. (Fig. 1E) The patient did not return to the hospital for review. Telephonic follow-up was conducted 1 month and 3 months after treatment and the patient reported stable vision in the right eye.

### 2.2 Patient 2

**Figure 2:**
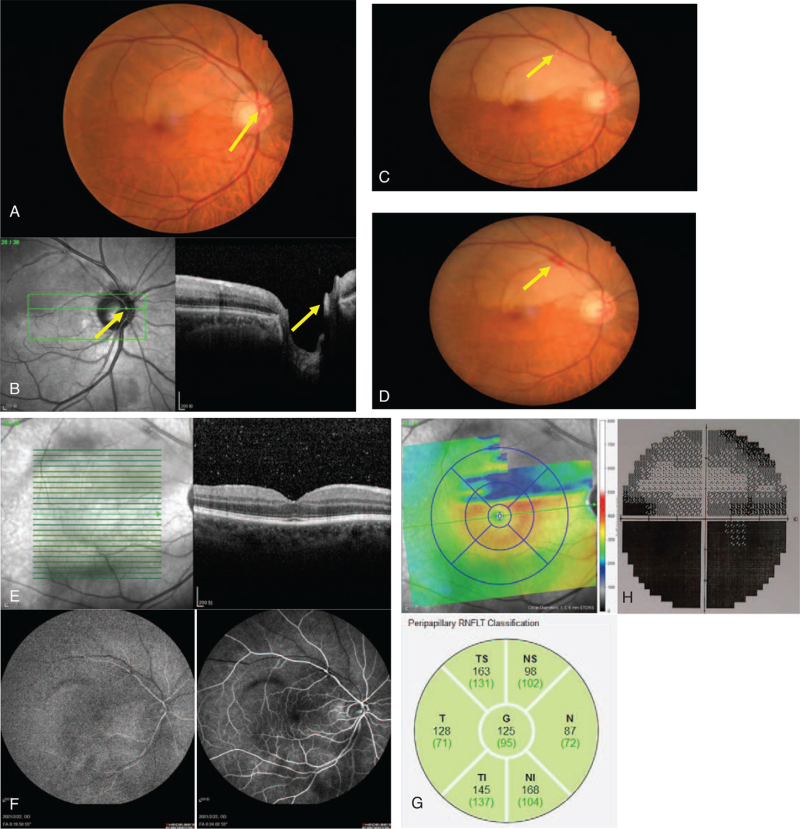
A: Color retinography showed superior hemi-retinal artery branch occlusion, retinal oedema in the superior hemi-retinal region, the visible embolus was located in the trunk of the upper branch of the central retinal artery on the surface of the optic papilla. B: OCT showed increased reflectivity and thickness of the inner retina in the surrounding superior hemi-retinal area, and Hyper-reflex signal in the lumen of the retinal artery. C: Color retinal photography showed superior hemi-retinal branch artery blood flow restoration, but residual embolus debris occluded the second stage branch of the superior-temporal retinal artery. D: Color retinal photography showed a small amount of linear bleeding and oedema was visible at the laser point. E: OCT showed reduced reflectivity in the inner layer of the retina. F: FAG showed the fill time of the affected superior temporal artery had backed to normal, and the fill time of the superior hemi-retinal vein had a delay of five seconds. G: OCT showed atrophy of the superior of retinal ganglion cells, the thickness of retinal nerve fibers around optic disc was normal. H: VF showed inferior arcuate scotoma. FAG = fluoresceine angiography, OCT = optical coherence tomography, VF = visual field.

A 65 years old male patient complained of a history of sudden blurry vision and inferior VF defect in his right eye which occurred 3 hours ago. At the time of ocular examination, his BCVA was 8/20 in the right eye and 20/20 in the left eye. IOP and slit-lamp examinations were normal in both eyes. The patient had no ocular history of disease, surgery, or trauma to either eye. However, the patient had a history of uncontrolled hyperlipidemia and carotid plaque. After thorough investigations including color retinography and OCT testing, the diagnosis was superior hemi-retinal artery branch occlusion with retinal oedema in the superior hemi-retinal region. The visible embolus was located in the trunk of the upper branch of the central retinal artery on the surface of the optic papilla (Fig. 2A). Additionally, increased reflectivity and thickness of the inner retina in the surrounding superior hemi-retinal area and Hyper-reflex signal in the lumen of the retinal artery were found through OCT (Fig. 2B). The fundus of the left eye was normal.

After dilating the blood vessels, lowering IOP, massaging the eyeball, and oxygen administration, the patient's right eye symptoms could not be relieved. After obtaining informed consent for TYE for BRAO, the TYE procedure was successfully completed, thus breaking the embolus and completely restoring blood flow. A 0.5 mJ pulse was initially delivered directly to the embolus for embolysis. At the same time, when the YAG laser penetrated the artery wall, a small amount of blood flowed out, and the bleeding stopped after proper pressure using the contact laser lens.

On the second day after laser treatment, the patient's right eye BCVA remained 8/20. Fundus examination and color retinal photography showed only a small portion of the embolus had been removed. Increased retinal edema, and the blocked retinal artery were observed. We performed a second laser treatment with pulse energy of 0.3 to 0.5 mJ. Starting from the proximal end of the embolus, the pulse power of the laser was used to break the embolus gradually until the embolus was removed as observed under the laser microscope and blood flow was restored. The broken embolus of the blood moved slowly to the farther end of the retinal artery.

After the second laser treatment, the patient's right eye BCVA improved to 16/20, fundus examination and color retinal photography showed superior hemi-retinal branch artery blood flow restoration, but residual embolus debris occluded the second stage branch of the superior-temporal retinal artery (Fig. 2C). A third laser treatment was performed, expecting to remove all embolus as far as possible at first with a lower energy (<0.5 mJ). Upon failure, laser energy increases from 0.5 to 0.6 mJ, attempting to breakdown the arterial wall, expecting to dislodge the embolus to the vitreous cavity.

Follow-up one week later the patient's right eye BCVA was still 16/20, color retinal photography showed a small amount of linear bleeding and oedema was visible at the laser point (Fig. 2D). OCT showed reduced reflectivity in the inner layer of the retina (Fig. 2E), and fluoresceine angiography showed the fill time of the affected superior temporal artery had backed to normal, and the fill time of the superior hemi-retinal vein had a delay of 5 seconds (Fig. 2F). OCT showed atrophy of the superior retinal ganglion cells, the thickness of retinal nerve fibers around optic disc was normal (Fig. 2G). VF showed inferior arcuate scotoma (Fig. 2H). One month later, through telephonic follow-up, the patient reported stable vision in the right eye.

## 3 Discussion

Numerous treatment modalities have been attempted in both CRAO and BRAO without much success including low-intensity photocoagulation, intravenous prostaglandin E1 infusion, and enhanced external counter pulsation.^[7]^ TYE was proposed for the treatment of in 2002. Sixty-one treated cases were reported in the world, 47 with BRAO and 14 with CRAO. In a weighted analysis vitreous/sub-retinal hemorrhage was estimated to occur in 54% of cases and required vitrectomy in 18% of cases.^[8–11]^ Unfortunately, TYE has not been recognized by the academic community due to the condition of equipment and the lack of treatment experience.

Selaru et al^[3]^ reported their experience with TYE or embolectomy in 19 patients (9 BRAO and 10 CRAO) over a 5-year period. All of the patients had immediate and dramatic restoration of retinal blood flow following TYE or embolectomy as documented by fluorescein angiography. Furthermore, they mentioned that they did not find a correlation between the duration of the RAO and vision recovery. Seven patients had vitreous hemorrhage at the time of the embolectomy. One patient developed a large subhyaloid hemorrhage in an area corresponding to the vascular supply of the occluded BRA. This resulted in disappearance of the emboli and immediate restoration of retinal blood flow.

They suggested that this technique might be beneficial in eyes with RAO where an embolus is visible.

Wang reported the use of TYE combined with urokinase thrombolysis is effective for reperfusion of the occluded retinal arteries and improving visual recovery in patients with visible emboli.^[12]^ Mason et al^[2]^ reported 5 patients with BRAO who were treated with TYE. They noted that all patients showed improvement in best-corrected visual acuity one day after TYE. Fluorescein angiography showed immediate and dramatic restoration in flow past the obstructed arteriole in all patients. Transluminal embolysis was performed with a Nd:YAG (Ellex, **UltraQ Reflex**, **AU**) laser (1064 nm). The advantage of using this machine is the possibility of focusing together the laser beam and the image of the slit-lamp perpendicularly on the center of the cornea, not just obliquely. Thus, using specific laser contact lenses, it is possible to focus the target areas along the posterior pole.

Throughout our cases we have used a Volk Idrees Mid Vitreous Direct contact lens. The focusing was performed perpendicularly on the embolus surface. The spot diameter was constant 4 to 8 μm in size and the power ranged from 0.3 to 0.6 mJ. Firing was done “shot by shot”. In each session there were between 2 and 10 shots fired. Potential complications regarding this technique are: retinal or vitreous hemorrhage, retinal breaks, choroidal neovascularization, epiretinal membranes.

Kocak et al^[13]^ report a case where a patient who had BRAO and the clinical efficacy of the TYE on BRAO with visible embolus. The power ranged from 2 to 6mJ, and led to the developed of a subhyaloid hemorrhage overlying the occluded vessel area.^[13]^ Our patients also had a minor preretinal hemorrhages, and the bleeding stopped after proper pressure using the contact laser lens. The hemorrhages were completely reabsorbed after 10 to 14 days.

With this methodology, part of the embolus could pass into the vitreous. We differ from the literature that has been reported by proposing new ideas. There are two things that can happen by choosing different energy levels: massage blood vessel massage (which does not damage the vessel wall) and blood vessel breakdown (which does destroy the vessel wall) depending on the location, hardness, and size of the embolus). The lowest energy threshold, <1 mJ, may be safer. An initial attempt to remove the embolus is usually made using a low energy level (<0.5 mJ). After performing TYE, photo disruption of the embolus could have supported the reflow of retinal blood through the recanalization of the embolus. For softer emboli, such as platelet fibrino or cholesterol ones, the laser impacts usually dislodged them downstream the vessel in smaller fragments, if the power is set properly and the focus is done correctly.^[12]^ In this case, the embolus can be removed through vascular YAG laser surgery.

We noted after the removal or reduction of the embolus there is an increase in the caliber of the arterioles during the follow-up period. After TYE, the retinal artery contracted in response to stimulation and recovered within a few hours. However, the synchrony of recirculation and restoration of vision immediately following TYE or embolectomy suggests a beneficial effect of this procedure. In light of our experience in the two cases, TYE can be taken into account and can be an option and feasible technique for restoring retinal blood flow and allowing for retinal recuperation and return of visual function in the majority of patients with BRAO. In all cases, improvement of visual function was observed and full restoration of blood flow was achieved. The absolute therapeutic role of TYE in BRAO is debatable as it is known that over 70% of cases can heal by themselves, but the efficiency of the technique is clear when speaking about the speed of emboli destruction and the recovery of blood flow.^[13]^

Despite the set of inherent risks of any surgical intervention, the moment of emboli destruction as close as possible to the onset of occlusion is decisive for regaining visual function. We emphasize the importance of individualized analysis of any BRAO patient in deciding the treatment. The time of meaningful treatment for RAO, is determinant as the sooner the treatment is performed, the better results are achieved, but even in prolonged occlusions (<8–10 days) with persistent macular edema TYE embolysis can help.^[12,13]^

Currently, we cannot prove the benefits of late TYE. It is very difficult to decide if the retina lesions are for good after one week of BRAO or there are still some viable retinal cells able to survive if the blood flow is restablished. In addition, we should pay attention to systemic diseases such as giant cell arteritis, hyperlipidemia, hypertension, diabetes, chylous blood, carotid thrombosis. Attention needs to be focused on carotid ultrasound, echocardiography, erythrocyte sedimentation rate and fever.

Examinations were performed to understand the presence and degree of stenosis of the common carotid artery. Orbital CDI was added to detect blood flow signals parallel to the optic nerve to understand the velocity and direction of blood flow around the eye and to determine, as far as possible, the compensatory flow supply and collateral circulation pattern of the intracranial circulation.

## 4 Conclusion

Worldwide experience with TYE is still limited, but the technique seems feasible when treating RAO caused by visible emboli on the optic disc surface and posterior pole of the fundus oculi. Upon the successful competition of the TYE procedure the embolus was removed completely, restoring the blood flow in the intraocular vessels and improving significantly the patients’ visual acuity. The most important thing is the risks TYE has must be weighed against the possibility of severe and permanent loss of vision secondary to retinal artery occlusions. This most certainly calls for random trials for precisely identifying the role of TYE in the treatment of retinal occlusion pathology.

## Acknowledgments

We would like to thank Editage (www.editage.com) for English language editing.

## Author contributions

**Data curation:** Ning An, Xi Zhang.

**Supervision:** Wei Gu.

**Writing – original draft:** Wei Zhang.

**Writing – review & editing:** Wei Gu, Xiaoyan Peng.
